# Supplementary material for: Optimal Synthesis of Novel Phosphonic Acid Modified Diatomite Adsorbents for Effective Removal of Uranium(VI) Ions from Aqueous Solutions
Source: Materials (Basel). 2023 Jul 26;16(15):5263. doi: 10.3390/ma16155263 (PMC10419580; doi:10.3390/ma16155263)
Supplement: Supplementary file 1 [file materials-16-05263-s001.zip › materials-2133250-supplementary.pdf]

Electronic Supporting Information

Optimal synthesis of novel phosphonic acid modified diatomite adsorbents for effective removal of uranium(VI) ions from aqueous solutions

Natalia Kobylinska[1], Oksana Dudarko[2], Agnieszka Gładysz-Płaska[3*], Valentyn Tertykh[2], Marek Majdan[3]

[1]A.V. Dumansky Institute of Colloid and Water Chemistry, National Academy of Science of Ukraine, 42, Akad. Vernadskogo blvd., Kyiv, 03142, Ukraine, nkobilinskaya@gmail.com (N.K.);
[2]Chuiko Institute of Surface Chemistry, National Academy of Science of Ukraine, 17 General Naumov Str., Kyiv, 03164, Ukraine, Tertykh@yahoo.com (V.T.); odudarko80@gmail.com (O.D.);
[3]Department of Inorganic Chemistry, Institute of Chemical Sciences, Faculty of Chemistry, Maria Curie-Sklodowska University, M. Curie- Sklodowska Sq. 2, 20-031 Lublin, Poland, majdan.marek8@gmail.com (M.M);
* Correspondence: agnieszka.gladysz-plaska@mail.umcs.pl. (A.G.); nkobilinskaya@gmail.com (N.K.)

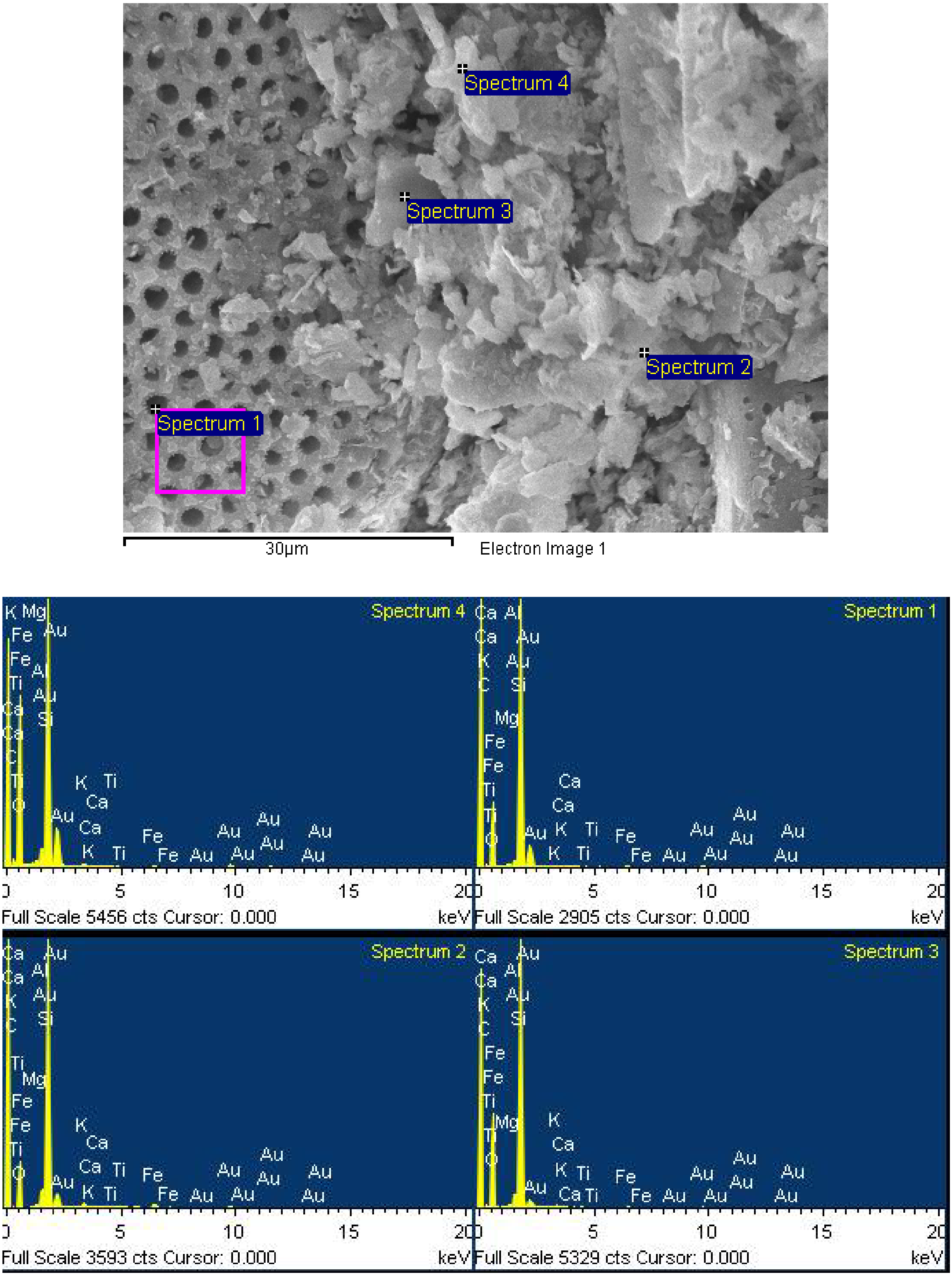

Figure S1. SEM image and EDX spectra of natural diatomite

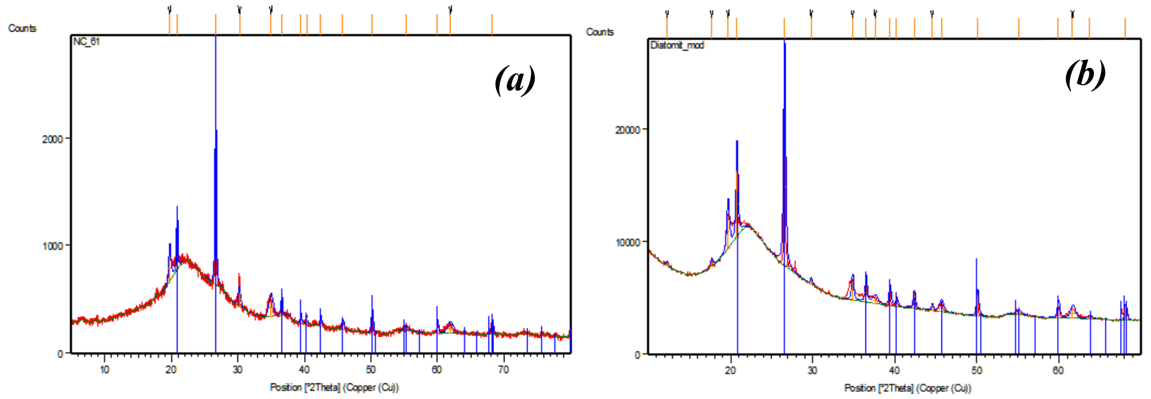

Figure S2. Powder XRD patterns of natural diatomite (***a***) and organo-diatomite (***b***) compared with reference materials (*quartz* – JCPDS, No 46-1045, *calcite* – JCPDS, No 05-0586 and *illite* – JCPDS, No 43-0685).

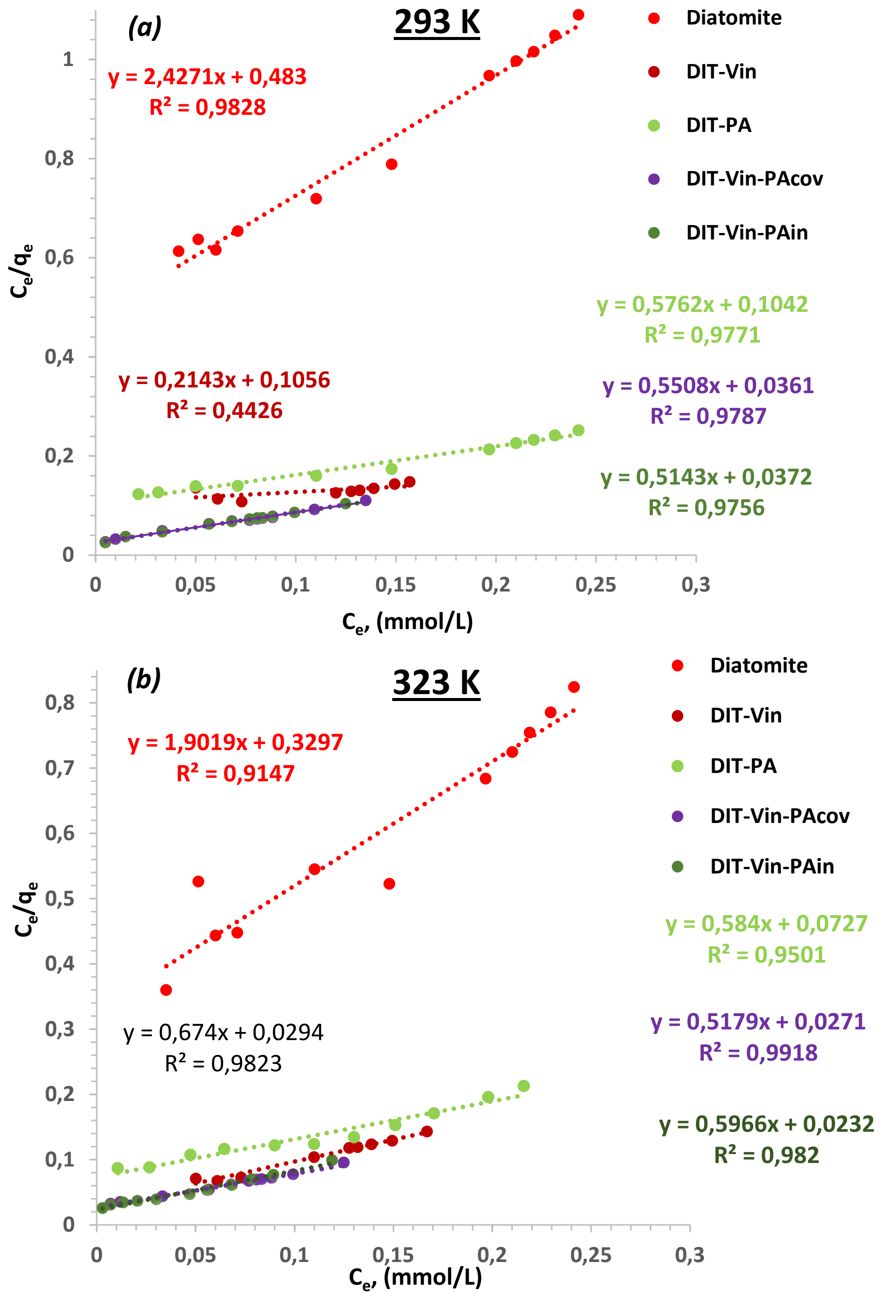

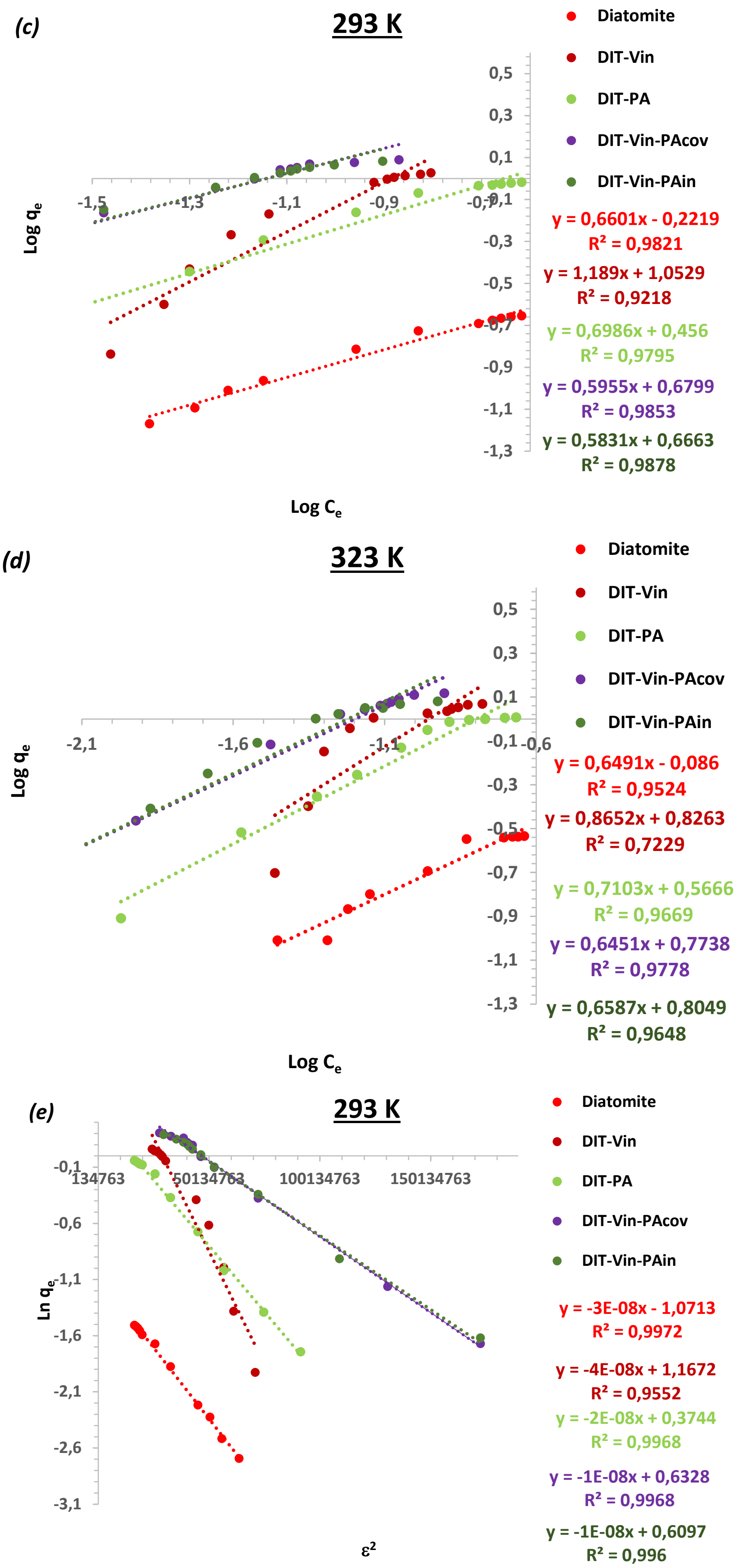
(c)
293 K
Log qe
Log Ce
Diatomite
DIT-Vin
DIT-PA
DIT-Vin-PAcov
DIT-Vin-PAin
y = 0,6601x - 0,2219
R² = 0,9821
y = 1,189x + 1,0529
R² = 0,9218
y = 0,6986x + 0,456
R² = 0,9795
y = 0,5955x + 0,6799
R² = 0,9853
y = 0,5831x + 0,6663
R² = 0,9878
(d)
323 K
Log qe
Log Ce
Diatomite
DIT-Vin
DIT-PA
DIT-Vin-PAcov
DIT-Vin-PAin
y = 0,6491x - 0,086
R² = 0,9524
y = 0,8652x + 0,8263
R² = 0,7229
y = 0,7103x + 0,5666
R² = 0,9669
y = 0,6451x + 0,7738
R² = 0,9778
y = 0,6587x + 0,8049
R² = 0,9648
(e)
293 K
Ln qe
ε²
Diatomite
DIT-Vin
DIT-PA
DIT-Vin-PAcov
DIT-Vin-PAin
y = -3E-08x - 1,0713
R² = 0,9972
y = -4E-08x + 1,1672
R² = 0,9552
y = -2E-08x + 0,3744
R² = 0,9968
y = -1E-08x + 0,6328
R² = 0,9968
y = -1E-08x + 0,6097
R² = 0,996

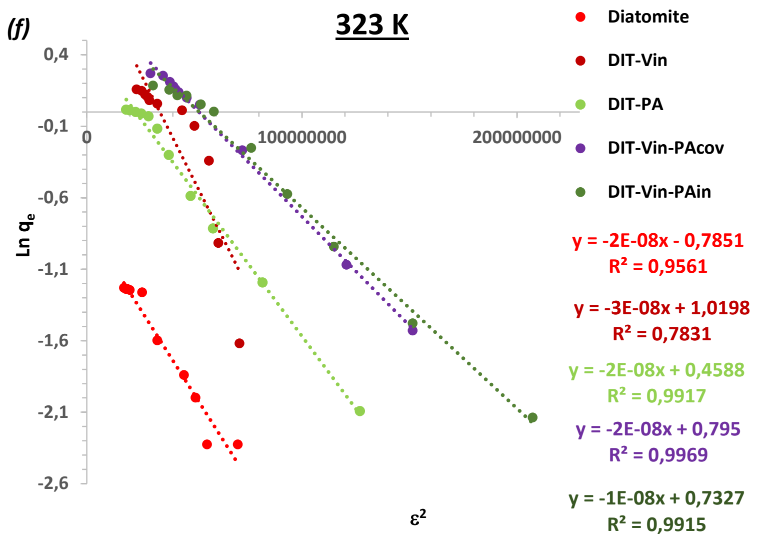

Figure S3. The linearized forms of the isotherms adsorption of U(VI) ions on obtained adsorbents at 293 K (*a, c* and *e*) and 323 K (*b, c* and *f*) according to Langmuir (*a, b*), Freundlich (*c, d*) and Dubinin-Radushkevich (*e, f*) models.

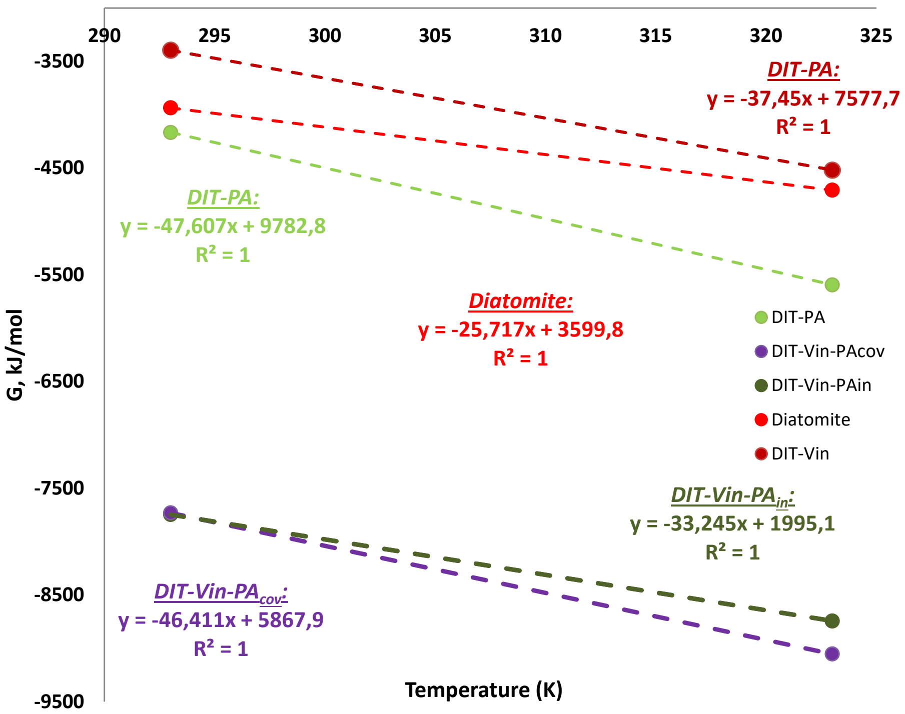

Figure S4. Effect of temperature on the Gibbs free energy for U(VI) ions adsorption on various adsorbents.
